# Production of extracellular superoxide and hydrogen peroxide by five marine species of harmful bloom-forming algae

**DOI:** 10.1093/plankt/fby043

**Published:** 2018-11-02

**Authors:** Julia M Diaz, Sydney Plummer, Carmelo Tomas, Catharina Alves-de-Souza

**Affiliations:** 1 Skidaway Institute of Oceanography, Department of Marine Sciences, University of Georgia, Savannah, GA, USA; 2 Algal Resources Collection, MARBIONC at CREST Research Park, University of North Carolina Wilmington, Wilmington, NC, USA

**Keywords:** reactive oxygen species, superoxide, hydrogen peroxide, *Pseudo-nitzschia*, *Karenia brevis*, *Aureococcus anophagefferens*

## Abstract

Harmful bloom-forming algae include some of the most prolific microbial producers of extracellular reactive oxygen species (ROS). However, the taxonomic diversity of ROS production, the underlying physiological mechanisms and ecophysiological roles of ROS cycling are not completely characterized among phytoplankton taxa that form harmful algal blooms (HABs). This study examines the extracellular production of the ROS superoxide and hydrogen peroxide by five marine HAB species: *Chattonella marina*, *Heterosigma akashiwo*, *Karenia brevis*, *Pseudo-nitzschia* sp. and *Aureococcus anophagefferens*. All species produced extracellular superoxide and hydrogen peroxide. Rates of ROS production per cell spanned several orders of magnitude and varied inversely with cell density, suggesting a potential signaling role for extracellular ROS. ROS production was also detected in the spent media of all cultures except *K. brevis*, indicating the presence of cell-free ROS-generating constituents, such as enzymes or metabolites, which could be further investigated as molecular targets for tracking ROS production in laboratory and field settings. Finally, ratios of superoxide to hydrogen peroxide production could not be accounted for by superoxide dismutation alone, except in the case of *K. brevis*, indicating a diversity of ROS production and degradation pathways that may ultimately help illuminate the functions of HAB-derived ROS.

## INTRODUCTION

The reactive oxygen species (ROS) superoxide and hydrogen peroxide are intermediates in the reduction of oxygen to water. These ROS are powerful oxidants and reductants that shape ecological interactions and biogeochemistry in aquatic environments. For example, ROS contribute to the cycling of carbon (Pullin *et al.*, 2004), transform vital trace metals such as iron (Rose, 2012) and regulate toxic elements like mercury (Siciliano *et al.*, 2002). Recognition has been growing that extracellular ROS production by plankton communities contributes substantially to aquatic ROS fluxes (Zinser, 2018). Indeed, extracellular ROS production has been documented in several microbial groups including heterotrophic bacteria (Diaz *et al.*, 2013), cyanobacteria (Rose *et al.*, 2008; Hansel *et al.*, 2016), diatoms (Kustka *et al.*, 2005; Schneider *et al.*, 2016), dinoflagellates (Saragosti *et al.*, 2010; Zhang *et al.*, 2016) and raphidophytes (Oda *et al.*, 1997; Portune *et al.*, 2010).

As a group, harmful bloom-forming algae exhibit the highest rates of extracellular ROS production observed among aquatic microorganisms. These ROS contribute to the toxic or noxious activity of several harmful algal bloom (HAB) taxa, such as raphidophytes (Oda *et al.*, 1992, 1997; Yang *et al.*, 1995; Kim *et al.*, 1999b) and the dinoflagellates *Margalefidinium polykrikoides* (Kim *et al.*, 1999a; Tang and Gobler, 2009b; Tang and Gobler, 2010) and *Alexandrium* spp. (Flores *et al.*, 2012; Mardones *et al.*, 2015). However, extracellular ROS production by these HAB species is not always linked to allelopathic or ichthyotoxic effects (Twiner *et al.*, 2001; Marshall *et al.*, 2003; Woo *et al.*, 2006; Tang and Gobler, 2009a). In the harmful raphidophyte *Chattonella marina*, extracellular ROS have also been implicated in metal nutrient acquisition (Garg *et al.*, 2007; Liu *et al.*, 2007) and autocrine growth promotion (Oda *et al.*, 1995), consistent with a range of other cell types (Saran, 2003; Buetler *et al.*, 2004; Rose *et al.*, 2005; Mittler *et al.*, 2011; Roe and Barbeau, 2014). Thus, extracellular ROS production by HAB species may have a myriad of impacts on aquatic ecology and biogeochemistry, which are not completely understood. Here, we explore the rates, cell density-dependent regulation and mechanisms of extracellular ROS production by five species of harmful bloom-forming algae, including the key taxa *Karenia brevis*, *Pseudo-nitzschia* sp. and *Aureococcus anophagefferens*, for which extracellular ROS production either has not been reported or not been quantified previously.

## MATERIALS AND METHODS

### Algal strains, culturing conditions and sampling

*C. marina* ARC260, *Heterosigma akashiwo* ARC114, *K. brevis* ARC5, and *Pseudo-nitzschia* sp. ARC447 were obtained from the Algal Resources Collection at the University of North Carolina Wilmington (www.algalresourcescollection.com). *A. anophagefferens* CCMP1984 was obtained from the National Center for Marine Algae and Microbiota, Bigelow Laboratories, East Boothbay, Maine. All cultures were maintained in L1 media (Guillard and Hargraves, 1993) at 23°C on a 14 h:10 h light:dark cycle (340 μmol photons m^−2^ s^−1^), with the following exceptions: *A. anophagefferens* was grown at 18°C, and *Pseudo-nitzschia* sp. was cultured in *f*/25 (Guillard and Ryther, 1962) at 18°C. All media were prepared using filtered (0.2 μm) natural seawater from the South Atlantic Bight. Phytoplankton growth was monitored by measuring *in vivo* chlorophyll fluorescence with an AquaFluor handheld fluorometer (Turner Designs). *C. marina* and *H. akashiwo* were counted live using a Multisizer 4e coulter counter (Beckman Coulter). All other cultures were preserved in 2% Lugol’s solution and enumerated under the microscope using a hemocytometer counting chamber (Karlson *et al.*, 2010). Cultures were sampled for ROS measurements within the first half of the 14-h daylight period during late-exponential growth, except *A. anophagefferens*, which was analyzed at the same point in the diel cycle but in early log phase. Cell abundances (cells mL^−1^) at the time of sampling were as follows: *C. marina* (1.4 × 10^4^), *H. akashiwo* (1.5 × 10^5^), *K. brevis* (5.3 × 10^3^), *Pseudo-nitzschia* sp. (1.0 × 10^4^), *A. anophagefferens* (2.1 × 10^5^).

A dilution series of each culture was prepared using spent media (i.e. cell-free filtrate). For all species except *A. anophagefferens*, the cell-free filtrate was prepared by gravity filtering an aliquot of each culture (5 μm, 47 mm) through a polycarbonate membrane (EMD Millipore) and subsequently syringe-filtering (0.2 μm). To generate cell-free filtrates of *A. anophagefferens*, cultures were centrifuged (3000 × g, 10 min, 4°C), decanted, and then syringe-filtered (0.2 μm). For all species, the original, light-adapted culture was then diluted into the filtrate at a ratio of 1:10 and 1:100. After preparation, culture dilution series and the cell-free filtrate were immediately analyzed for extracellular ROS production under the conditions specified below for each ROS. After ROS measurements, microscopic inspection of the diluted and undiluted cultures confirmed that cell morphology and motility remained unaltered by sample handling and analysis.

### Superoxide

Superoxide production rates were quantified based on the specific reaction between superoxide and the chemiluminescent probe methyl *Cypridina* luciferin analog (MCLA), to which cell membranes are impermeable. To avoid breakage of the relatively large, fragile cells examined in this study, superoxide production was quantified by adding MCLA to cell suspensions in a microplate assay (Godrant *et al.*, 2009), rather than using a common flow injection analysis method that has been applied to measure extracellular superoxide production by smaller bacteria and phytoplankton cells immobilized on a filter (Diaz *et al.*, 2013; Schneider *et al.*, 2016; Zhang *et al.*, 2016). Culture dilution series and cell-free filtrates were analyzed in replicate in a white 96-well plate with the addition of superoxide dismutase (SOD, 40 kU L^−1^, to account for the autooxidation of MCLA), each of three xanthine oxidase calibration standards (XO, 5, 10 and 50 mU L^−1^), or no addition. To complex metals that otherwise lower the lifetime and detectability of superoxide, diethylenetriaminepentaacetic acid (DTPA, 100 μmol L^−1^) was added to all wells, in addition to xanthine (X, 50 μmol L^−1^) and MCLA (5.7 μmol L^−1^). Chemiluminescence was measured at all wavelengths every 3.5 min for 1 h with a 1 s acquisition time per well using a SpectraMax M series multimode plate reader (Molecular Devices). This incubation period is in the acceptable range for this method given the concentrations of X and XO used (Godrant *et al.*, 2009). Cultures remained in the dark (0 μmol photons m^−2^ s^−1^) within the plate reader during the 1 h analysis.

Superoxide production rates were calculated as follows. At each time point, each unamended and XO-amended sample was corrected for the baseline chemiluminescence measured in the presence of SOD, and then averaged across all time points. The average SOD-corrected signals from unamended samples were then converted to XO equivalents using the internal XO calibration curve for each sample. Finally, the XO standard (30 U L^−1^) was calibrated in the cell-free filtrate (50 μmol L^−1^ X, 100 μmol L^−1^ DTPA) in the presence of nitroblue tetrazolium (NBT, 100 μmol L^−1^), based on the rate of monoformazan production (MF, molar extinction coefficient = 12 800 L mol^−1^ cm^−1^, in a 1:2 molar ratio of MF:superoxide; (Bielski *et al.*, 1980)) in the presence and absence of SOD (40 kU L^−1^). The superoxide production rate in the filtrate was subtracted from all diluted and undiluted culture samples. In subsequent experiments using an identical approach, background rates of superoxide production were determined in sterile (virgin) L1 and *f*/25 media, which were used to correct the superoxide production rates of cell-free filtrates. Corrected superoxide production rates in cell-free filtrates were normalized to the original cell density in the undiluted culture at the time of sampling. All chemicals were obtained from Millipore Sigma.

### Hydrogen peroxide

Hydrogen peroxide production rates were measured from each culture at the same time as superoxide production rates. The hydrogen peroxide analysis was based on the reaction between hydrogen peroxide and the colorimetric probe Ampiflu™ Red (AR), which is catalyzed extracellularly by horseradish peroxidase (HRP). Culture dilution series and cell-free filtrates were analyzed in replicate in a clear 96-well plate with the addition of the hydrogen peroxide-degrading enzyme catalase (10 mg L^−1^; to account for the autooxidation of AR), each of four concentrations of hydrogen peroxide (10, 100, 700, 5000 nmol L^−1^), or no addition. Hydrogen peroxide standards were prepared from a primary stock solution made by diluting 2 μL of 30% hydrogen peroxide into 4 mL of ultrapure water, which was calibrated by measuring its absorbance at 240 nm and applying the molar extinction coefficient of hydrogen peroxide at this wavelength, 38.1 L mol^−1^ cm^−1^ (Miller and Kester, 1988). AR and HRP were added at final concentrations of 18 μmol L^−1^ and 0.4 kU L^−1^, respectively. Cultures were incubated under ambient low light conditions (~5 μmol photons m^−2^ s^−1^) for up to 4 h, and absorbance was measured at 530 nm and 700 nm once an hour using a SpectraMax M series multimode plate reader (Molecular Devices).

Hydrogen peroxide production rates were calculated as follows. First, absorbance at 700 nm was subtracted from the absorbance at 530 nm in every well at each time point. Next, at each time point, each unamended and hydrogen peroxide-amended sample was corrected for the baseline absorbance measured in the presence of catalase. Then, each catalase-corrected signal from the unamended samples was converted to hydrogen peroxide concentration using the internal hydrogen peroxide calibration curve for each sample. Finally, the increase in hydrogen peroxide in each unamended sample over time was quantified with simple linear regression (R typically > 0.95). The hydrogen peroxide production rate in the cell-free filtrate was subtracted from all diluted and undiluted culture samples. In subsequent experiments using an identical procedure, background rates of hydrogen peroxide production were determined in sterile (virgin) L1 and *f*/25 media, which were used to correct the hydrogen peroxide production rates of cell-free filtrates. Corrected hydrogen peroxide production rates in cell-free filtrates were normalized to the original cell density in the undiluted culture at the time of sampling. All chemicals were obtained from Millipore Sigma.

### Statistical analysis

Statistical analyses were conducted in JMP Pro (version 13.0). Potential monotonic relationships between cell density and cell-normalized ROS production rates were determined by calculating Spearman’s rank correlation coefficient. Average ratios of superoxide to hydrogen peroxide production were analyzed using a one-sample *t*-test of the population mean.

## RESULTS

### Superoxide

**Fig. 1.**
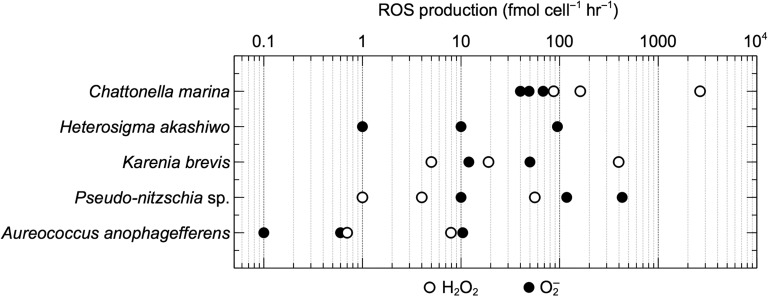
Cell-associated rates of extracellular superoxide (O_2_^−^) and hydrogen peroxide (H_2_O_2_) production by the organisms examined in this study.

**Fig. 2.**
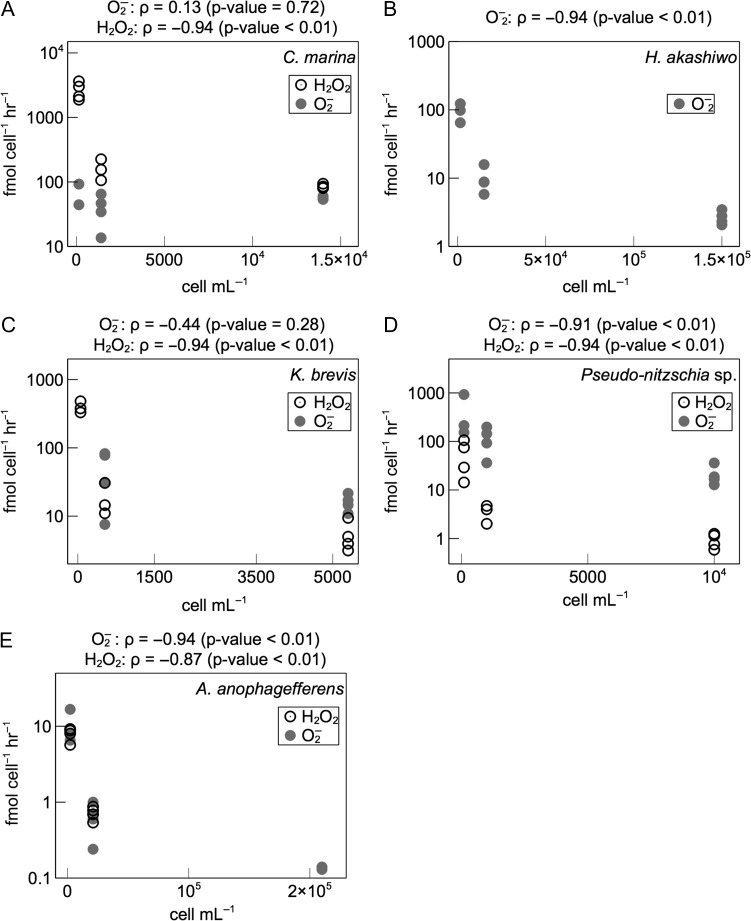
Cell density dependence of extracellular superoxide (O_2_^−^) and hydrogen peroxide (H_2_O_2_) production. Spearman’s rank correlation coefficient (*ρ*) and associated *P*-value describe the degree of monotonicity in the relationship between cell-normalized superoxide production rates and cell density.

Rates of extracellular superoxide production measured over a broad range of cell densities of *C. marina*, *H. akashiwo*, *K. brevis*, *Pseudo-nitzschia* sp. and *A. anophagefferens* were corrected for superoxide production rates in cell-free filtrates. These corrected rates therefore reflect cell-associated superoxide production. Cell-associated superoxide production rates exhibited substantial inter- and intraspecific variability (Fig. 1; Table I). Maximum rates (average ± SE) were observed in *Pseudo-nitzschia* sp. (431 ± 248 fmol cell^−1^ h^−1^; *n* = 3), followed by *H. akashiwo* (95 ± 15 fmol cell^−1^ h^−1^; *n* = 3), *C. marina* (68 ± 24 fmol cell^−1^ h^−1^; *n* = 2), *K. brevis* (50 ± 18 fmol cell^−1^ h^−1^; *n* = 4), and finally *A. anophagefferens* (10 ± 3 fmol cell^−1^ h^−1^; *n* = 3). Cell-associated superoxide levels remained below detection in only one case, which was *K. brevis* at the lowest cell density (~50 cells mL^−1^). In all species except *C. marina*, cell-normalized superoxide production rates increased with decreasing cell density, which was statistically significant (*P* < 0.01) in all species except *K. brevis* (Fig. 2). With 10-fold and 100-fold decreases in cell density, cell-normalized superoxide production increased by ~4–12 times and ~45 times, respectively, by *Pseudo-nitzschia* sp.; ~10 times and ~100 times, respectively, by *H. akashiwo*; and ~15 times and ~200 times, respectively, by *A. anophagefferens*. With a 10-fold decrease in cell density, cell-normalized superoxide production increased by ~4 times in *K. brevis*.

**Table I:** Extracellular superoxide (O_2_^−^) and hydrogen peroxide (H_2_O_2_) production by the organisms examined in this study

Species	Strain	Dilution level	cells mL^−1^	ROS Production	
				fmol cell^−1^ h^−1^	
				O_2_^−^	H_2_O_2_
*Chattonella marina*	ARC260	1:1	14 000	49 ± 1 (*n* = 4)	87 ± 3 (*n* = 4)
		1:10	1400	40 ± 11 (*n* = 4)	162 ± 35 (*n* = 3)
		1:100	140	68 ± 24 (*n* = 2)	2658 ± 408 (*n* = 4)
		Filtrate	0	5 ± 1 (*n* = 4)	12 ± 1 (*n* = 4)
*Heterosigma akashiwo*	ARC114	1:1	150 000	0.9 ± 0.3 (*n* = 4)	BD
		1:10	15 000	10 ± 2 (*n* = 4)	BD
		1:100	1500	95 ± 15 (*n* = 3)	BD
		Filtrate	0	1.5 ± 0.2 (*n* = 4)	1.2 ± 0.1 (*n* = 4)
*Karenia brevis*	ARC5	1:1	5300	12 ± 2 (*n* = 4)	5 ± 1 (*n* = 4)
		1:10	530	50 ± 18 (*n* = 4)	19 ± 6 (*n* = 3)
		1:100	53	BD	397 ± 45 (*n* = 3)
		Filtrate	0	BD	BD
*Pseudo-nitzschia* sp.	ARC447	1:1	10 000	10 ± 9 (*n* = 2)	0.9 ± 0.2 (*n* = 4)
		1:10	1000	118 ± 35 (*n* = 4)	4 ± 1 (*n* = 3)
		1:100	100	431 ± 248 (*n* = 3)	56 ± 21 (*n* = 4)
		Filtrate	0	24 ± 2 (*n* = 4)	BD
*Aureococcus anophagefferens*	CCMP1984	1:1	210 000	0.1 ± 0.1 (*n* = 4)	BD
		1:10	21 000	0.6 ± 0.2 (*n* = 4)	0.7 ± 0.1 (*n* = 4)
		1:100	2100	10 ± 3 (*n* = 3)	8 ± 1 (*n* = 4)
		Filtrate	0	0.1 ± 0.0 (*n* = 4)	0.1 ± 0.0 (*n* = 4)

The ROS production rates in cell-free filtrates were normalized to the original cell density in the undiluted (1:1) culture at the time of sampling. Mean ROS production rates ± standard error of the mean are provided. BD, below detection.

Superoxide production rates in cell-free filtrates were corrected for the rates of superoxide production in sterile media. After normalizing to the original (undiluted) cell density, the corrected superoxide production rates (average ± SE) in the cell-free filtrates were 24 ± 2 fmol cell^−1^ h^−1^ (*Pseudo-nitzschia* sp.; *n* = 4), 5 ± 1 fmol cell^−1^ h^−1^ (*C. marina*; *n* = 4), 1.5 ± 0.2 fmol cell^−1^ h^−1^ (*H. akashiwo*; *n* = 4), and 0.1 ± 0.0 fmol cell^−1^ h^−1^ (*A. anophagefferens*; *n* = 4). Superoxide production in sterile media was sufficient to explain the generation of hydrogen peroxide in cell-free filtrates of *K. brevis*.

### Hydrogen peroxide

Like superoxide, rates of extracellular hydrogen peroxide production measured over a broad range of cell densities of *C. marina*, *K. brevis*, *Pseudo-nitzschia* sp. and *A. anophagefferens* were corrected for hydrogen peroxide production rates in cell-free filtrates. These corrected rates therefore reflect cell-associated hydrogen peroxide production. Cell-associated hydrogen peroxide production rates exhibited a wide range of variation within and between species (Fig. 1; Table I). Hydrogen peroxide production remained below detection in undiluted *A. anophagefferens* and in all *H. akashiwo* samples, except the *H. akashiwo* cell-free filtrate. Maximum rates of hydrogen peroxide production (average ± SE) revealed *C. marina* as the most prolific hydrogen peroxide producer (2658 ± 408 fmol cell^−1^ h^−1^; *n* = 4), followed by *K. brevis* (397 ± 45 fmol cell^−1^ h^−1^; *n* = 3), *Pseudo-nitzschia* sp. (56 ± 21 fmol cell^−1^ h^−1^; *n* = 4), and finally, *A. anophagefferens* (8 ± 1 fmol cell^−1^ h^−1^; *n* = 4). Cell-normalized hydrogen peroxide production rates increased with decreasing cell density, which was statistically significant (*P* < 0.01) in all species (Fig. 2). With 10-fold and 100-fold decreases in cell density, cell-normalized hydrogen peroxide production increased by ~4–16 times and ~60 times, respectively, by *Pseudo-nitzschia* sp.; ~2–16 times and ~31 times, respectively, by *C. marina*; and ~3–21 times and ~74 times, respectively, by *K. brevis*. Similarly, a 10-fold decrease in cell density resulted in an ~11-fold increase in cell-normalized hydrogen peroxide production by *A. anophagefferens*.

Hydrogen peroxide production rates in cell-free filtrates were corrected for the rates of hydrogen peroxide production in sterile media. After normalizing to the original (undiluted) cell density, the corrected rates of hydrogen peroxide production (average ± SE) in the cell-free filtrates were 12 ± 1 fmol cell^−1^ h^−1^ (*C. marina*; *n* = 4), 1.2 ± 0.1 fmol cell^−1^ h^−1^ (*H. akashiwo*; *n* = 4), and 0.1 ± 0.0 fmol cell^−1^ h^−1^ (*A. anophagefferens*; *n* = 4). Hydrogen peroxide production in sterile media was sufficient to explain the generation of hydrogen peroxide in cell-free filtrates of *K. brevis* and *Pseudo-nitzschia* sp.

### Comparison of superoxide and hydrogen peroxide production rates

**Fig. 3.**
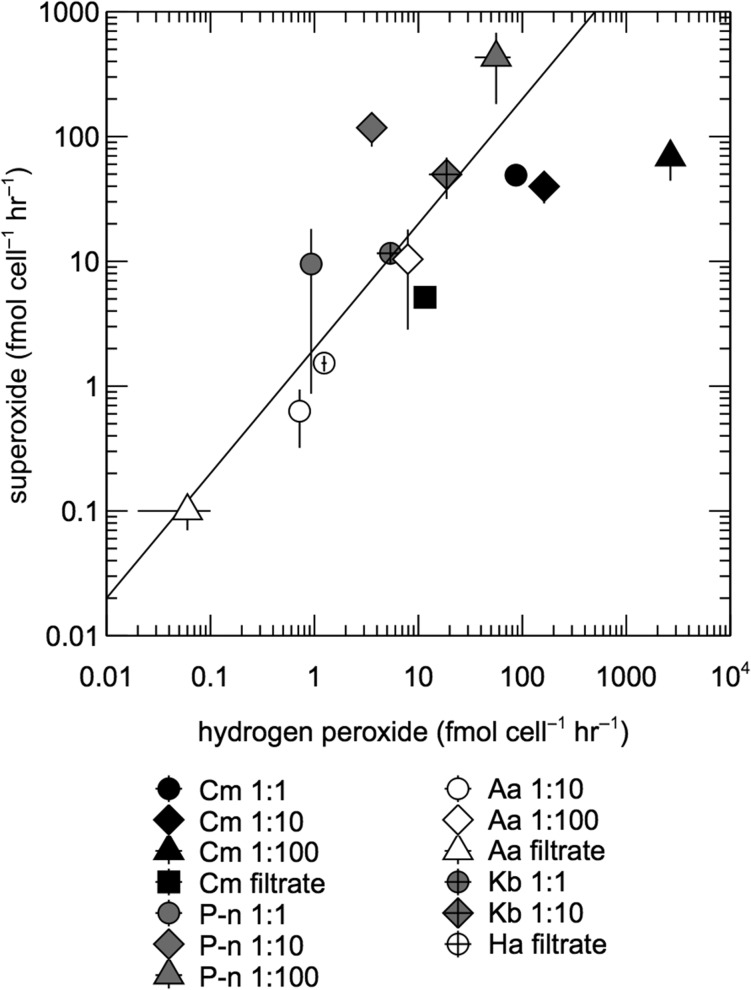
Comparison of superoxide and hydrogen peroxide production rates in diluted cell suspensions and cell-free filtrates. *C. marina* (Cm), *Pseudo-nitzschia* sp. (P-n), *A. anophagefferens* (Aa), *K. brevis* (Kb), and *H. akashiwo* (Ha). Error bars represent the standard error of the mean of biological replicates. The diagonal line represents the 2:1 molar ratio of superoxide to hydrogen peroxide production expected from the dismutation of superoxide.

A range of superoxide to hydrogen peroxide production ratios (P_O_2_−_:P_H_2___O_2__) were observed in the organisms tested here (Fig. 3), which reveal underlying dynamics of each ROS. For example, the complete reduction of superoxide to hydrogen peroxide yields P_O_2_−_:P_H_2___O_2__ = 1. The self-reaction of two moles of superoxide via dismutation produces one mole of hydrogen peroxide (P_O_2_−_:P_H_2___O_2__ = 2), while the complete oxidation of superoxide gives rise to no hydrogen peroxide, leading to P_O_2_−_:P_H_2___O_2__ > 2. *K. brevis* produced ROS at a ratio consistent with superoxide dismutation (P_O_2_−_:P_H_2___O_2__ = 2–3). However, *A. anophagefferens* (P_O_2_−_:P_H_2___O_2__ = ~1; *P* < 0.01), *H. akashiwo* (P_O_2_−_:P_H_2___O_2__ = ~1; *P*«0.01) and *C. marina* (P_O_2_−_:P_H_2___O_2__ = 0.03–0.6; *P*«0.01) produced more hydrogen peroxide than expected from superoxide dismutation alone. On average, *Pseudo-nitzschia* sp. produced less hydrogen peroxide expected from superoxide dismutation (P_O_2_−_:P_H_2___O_2__ = 8–30), which was statistically significant at the 1:10 and 1:100 dilution levels (*P* < 0.05), but not in the undiluted case (*P* = 0.07), due to the high level of variability in superoxide production rates measured in the undiluted culture (relative standard deviation >100%).

## DISCUSSION

In this study, the rates, cell density-dependent regulation and mechanisms of extracellular ROS production were explored in five marine species of harmful bloom-forming algae, including the key taxa *K. brevis*, *Pseudo-nitzschia* sp. and *A. anophagefferens*. Previous work has documented the ability of *K. brevis* to generate extracellular superoxide based on relative chemiluminescence (Marshall *et al.*, 2005a; Mooney *et al.*, 2011). Yet no other prior reports of extracellular ROS production by *K. brevis*, *Pseudo-nitzschia* sp., or *A. anophagefferens* could be found. Therefore, this study broadens the diversity of harmful algal species known to generate extracellular hydrogen peroxide and superoxide.

### Rates of ROS production by intact cells

Consistent with literature observations, *C. marina* was the most prolific ROS producer observed in this study, followed by intermediate-level ROS producers *H. akashiwo*, *K. brevis* and *Pseudo-nitzschia* sp., and finally *A. anophagefferens*. Although ROS production by *A. anophagefferens* was much lower than the other species examined, superoxide production rates by *A. anophagefferens* were comparable to *Synechococcus* sp., which has been shown to represent a substantial potential source of ROS in the marine environment (Rose *et al.*, 2008). Cell size is directly related to ROS production and thus drives substantial interspecific differences in extracellular ROS production rates (Oda *et al.*, 1997; Marshall *et al.*, 2005a; Diaz *et al.*, 2013). Indeed, *A. anophagefferens* (a ~2–5 μm cell) is much smaller than any other species examined in this study, and the relatively lower ROS production rates by this organism are to be expected. *A. anophagefferens* was also the only species in this study that was analyzed in early exponential phase, as opposed to late-exponential phase. Although the growth phase dependence of extracellular ROS production by *A. anophagefferens* is not known, biomass-normalized rates of extracellular ROS production decline with age in batch cultures of several marine phytoplankton, including *C. marina* (Oda *et al.*, 1995; Kawano *et al.*, 1996; Garg *et al.*, 2007), *Chattonella antiqua* (Portune *et al.*, 2010), *H. akashiwo* (Skeen *et al.*, 2004; Portune *et al.*, 2010) and *M. polykrikoides* (Kim *et al.*, 1999a). If the same is true for *A. anophagefferens*, then the rates measured in this study may be at the upper end of the range for this species, which is further consistent with the large interspecific differences in ROS production observed here.

Production rates of extracellular superoxide and hydrogen peroxide reported in this study are lower than previously documented rates of extracellular ROS production by HAB taxa (Diaz and Plummer, 2018), even compared to observations generated using the same or similar techniques on the same species (Dorantes-Aranda *et al.*, 2013; Mardones *et al.*, 2015). Because superoxide and hydrogen peroxide were measured by independent methods herein, the relatively low production rates measured for these ROS corroborate each other. Indeed, it is simpler to presume that the relatively low rates for both ROS reflect a shared biological explanation, rather than separate technical shortcomings of each method. The discrepancies between literature values and the rates measured in this study may be related to a number of factors. For example, ROS production can be affected by growth phase (Kawano *et al.*, 1996; Kim *et al.*, 1999a; Skeen *et al.*, 2004; Garg *et al.*, 2007; Portune *et al.*, 2010), cell density (Yang *et al.*, 1995; Twiner and Trick, 2000; Kim *et al.*, 2002; Marshall *et al.*, 2005b; Dorantes-Aranda *et al.*, 2015) and light exposure (Kim *et al.*, 1999a; Dorantes-Aranda *et al.*, 2013). Substantial intraspecific variability in extracellular ROS production has also been documented (Ishimatsu *et al.*, 1996; Oda *et al.*, 1997; Portune *et al.*, 2010; Dorantes-Aranda *et al.*, 2013; Mardones *et al.*, 2015), with extracellular superoxide production by different strains of *C. marina* varying up to 6-fold (Band-Schmidt *et al.*, 2012). Consistent with this intraspecific variability, *C. marina* ARC260 produced extracellular superoxide at an average rate of 52 fmol cell^−1^ hr^−1^ in this study, which was ~6–9 times lower than another strain, *C. marina* CMDE01, during the same late-exponential growth stage in a previous investigation (Garg *et al.*, 2007).

Cultures were not axenic. However, assuming bacterial abundance did not exceed 10^6^ cell mL^−1^, which is consistent with the lack of visible bacterial growth in the cultures, the contribution of bacterial associates would have been less than 0.2% (median) or 8% (maximum) of total ROS fluxes measured, based on previously determined rates of bacterial extracellular ROS production (Gonzàlez-Flecha and Demple, 1995; Diaz *et al.*, 2013). Because ROS measurements were conducted in the dark or under low light conditions below the compensation irradiances of the cultures (Wilson and Collier, 1955; Aldrich, 1962; Eng-Wilmot *et al.*, 1977; Yamaguchi *et al.*, 1991; Milligan and Cosper, 1997; El-Sabaawi and Harrison, 2006; Shikata *et al.*, 2008; Smayda, 2008), another potential concern is that oxygen concentrations could decrease due to respiration and potentially become rate-limiting on the production of extracellular ROS. However, ROS data did not appear to be limited by oxygen consumption because hydrogen peroxide concentrations increased linearly throughout incubations (*R* > 0.95), and superoxide chemiluminescence signals did not attenuate significantly over time. Moreover, the sustained production of superoxide for 1 h in the dark indicates the presence of non-photosynthetic pathways for superoxide generation. In agreement with these results, extracellular superoxide production by *C. marina* and *H. akashiwo* could not be quenched by the photosynthetic inhibitor dichlorophenyldimethylurea (DCMU) in a previous study (Oda *et al.*, 1998).

### The effect of cell density on ROS production

Cell-normalized superoxide and hydrogen peroxide production by all species varied inversely with 10- and 100-fold changes in cell density, except superoxide production by *C. marina* and *K. brevis*. Inverse relationships between cell density and cell-normalized ROS production have been observed previously in harmful algae (Twiner and Trick, 2000; Kim *et al.*, 2002; Marshall *et al.*, 2005b) and the colonial marine cyanobacterium *Trichodesmium* spp. (Hansel *et al.*, 2016). Such results suggest a cell density-dependent signaling role for ROS production in a variety of phytoplankton species, as outlined in a prior study. (Hansel *et al.*, 2016).

A number of potential artifacts are possible with the use of MCLA in cell suspensions, however, which could lead to the underestimation of superoxide at high cell densities. For example, MCLA chemiluminescence may be absorbed by chlorophyll and quenched via binding of the probe to cell membranes (Godrant *et al.*, 2009). These potential artifacts cannot be ruled out here, and thus, the current results must be interpreted with caution. However, using the same technique and approximately similar cell densities of *C. marina*, Dorantes-Aranda *et al.* (2013) reported much higher rates of superoxide production by this species. Thus, cell density may not be the predominant factor determining MCLA chemiluminescence in cell suspensions of *C. marina*. Moreover, because superoxide and hydrogen peroxide dynamics are coupled (e.g. superoxide gives rise to hydrogen peroxide via dismutation), the inverse relationship between cell-normalized hydrogen peroxide production and cell density supports the conclusion that the cell density dependence of superoxide production has a physiological basis.

### Cell-free ROS production

In addition to cell-associated ROS production, the results from this study demonstrate ROS generation in the cell-free filtrate of several species, which could not be accounted for with sterile media controls. For example, *C. marina*, *H. akashiwo* and *A. anophagefferens* exhibited superoxide and hydrogen peroxide production in the cell-free filtrate, while *Pseudo-nitzschia* sp. cell-free filtrates only generated superoxide. Any cell-free ROS production by *K. brevis*, if present, remained below detection. These results point to the production of ROS by cell-free constituents (e.g. enzymes or metabolites) in the spent media, consistent with previous observations from *C. marina* (Kim *et al.*, 2000; Li *et al.*, 2015), the diatom *Phaeodactylum tricornutum* (Schneider *et al.*, 2016), and a marine bacterium belonging to the Roseobacter clade of Alphaproteobacteria (Andeer *et al.*, 2015).

### Comparison of superoxide and hydrogen peroxide production

The underlying dynamics of superoxide and hydrogen peroxide can be inferred from the ratio of superoxide to hydrogen peroxide production. Here, comparisons of superoxide and hydrogen peroxide production were drawn from cultures of varying cell density, as well as cell-free filtrates. These comparisons were limited when superoxide and/or hydrogen peroxide levels were below detection (Table I). For hydrogen peroxide measurements, cultures were incubated in low ambient light (~5 μmol photons m^−2^ s^−1^), while superoxide measurements were conducted in the dark. Although photosynthesis may play a role in extracellular hydrogen peroxide production (Diaz and Plummer, 2018), the ambient light was likely below the compensation point for all species (Wilson and Collier, 1955; Aldrich, 1962; Eng-Wilmot *et al.*, 1977; Yamaguchi *et al.*, 1991; Milligan and Cosper, 1997; El-Sabaawi and Harrison, 2006; Shikata *et al.*, 2008; Smayda, 2008) and therefore would have probably supported only minimal rates of photosynthesis, which is comparable to the conditions during superoxide analysis.

ROS measurements from each species generally reflected a consistent relationship between superoxide and hydrogen peroxide production across all levels of cell density and the cell-free filtrate. In *K. brevis*, the disproportionation of superoxide (e.g. by the enzyme superoxide dismutase and/or uncatalyzed dismutation) can completely explain values of P_O_2_−_:P_H_2___O_2__, suggesting that extracellular hydrogen peroxide is only produced through a superoxide intermediate and that self-reaction is the predominant fate for superoxide. Comparable results were recently obtained for the diatom *Thalassiosira oceanica* (Schneider *et al.*, 2016), which also produced superoxide and hydrogen peroxide in a ratio consistent with superoxide dismutation. In contrast, low P_O_2_−_:P_H_2___O_2__ ratios suggest that additional sources of extracellular hydrogen peroxide besides superoxide dismutation must exist in cultures of *H. akashiwo*, *A. anophagefferens*, and *C. marina*, similar to previous results from *Thalassiosira pseudonana* (Schneider *et al.*, 2016). These sources may include the complete reduction of superoxide to hydrogen peroxide, direct enzymatic reduction of oxygen to hydrogen peroxide and the passive diffusion of hydrogen peroxide out of the cell. Indeed, extracellular superoxide and hydrogen peroxide production by *C. marina* appear to be decoupled according to previous research, with intracellular sources accounting for the majority of extracellular hydrogen peroxide and cell-surface oxidoreductase activity generating most of the extracellular superoxide (Oda *et al.*, 1997; Kim *et al.*, 2007). Finally, *Pseudo-nitzschia* sp. generated less hydrogen peroxide than expected from the dismutation of superoxide, similar to recent reports of *Thalassiosira weissflogii* (Schneider *et al.*, 2016). Although these results from *Pseudo-nitzschia* sp. were not always statistically significant, they suggest the presence of constituents that can oxidize superoxide and therefore prevent its reduction to hydrogen peroxide.

## CONCLUSIONS

All HAB species examined in this study produced extracellular superoxide and hydrogen peroxide, and cell-associated ROS production rates ranged several orders of magnitude within and between organisms. The potential role(s) of these extracellular ROS in the ecophysiology of *K. brevis*, *Pseudo-nitzschia* sp. and *A. anophagefferens* should now be considered. For example, the inverse relationship between cell-normalized ROS production and cell density suggests a population-dependent signaling role for these ROS, as previously suggested (Marshall *et al.*, 2005b; Hansel *et al.*, 2016), which transcends the common assumption that ROS are predominantly cytotoxic agents. Indeed, toxicity and signaling are not necessarily mutually exclusive functions, which is consistent with ROS playing a diversity of roles in HABs (Diaz and Plummer, 2018). Furthermore, an understanding of the substrates with which ROS react will improve knowledge of their biogeochemical and ecological functions. For example, superoxide has previously been implicated in reactions with polyunsaturated fatty acids (PUFAs), which yield toxic lipid oxidation products (Arzul *et al.*, 1998; Kim *et al.*, 1999a; Jenkinson and Arzul, 2001; Marshall *et al.*, 2003, 2005b; Mardones *et al.*, 2015). Similarly, results from this study revealed that a wide variety of reactions are likely involved in the production and degradation of extracellular superoxide and hydrogen peroxide in the species examined. For example, superoxide rapidly reacts with itself to produce hydrogen peroxide, which may fully explain ROS dynamics in *K. brevis*, but other, yet unknown oxidants are likely present in *Pseudo-nitzschia* sp., while additional reductants may exist in *A. anophagefferens* and *H. akashiwo*. The production of ROS observed in spent media points to cell-free constituents (e.g. enzymes and/or metabolites) that can be developed as molecular targets for tracking extracellular ROS production. Such tools may be especially useful in the field, where ROS may be rapidly cycled and difficult to detect by direct chemical methods.

## References

[fby043C1] Aldrich D. V. (1962) Photoautotrophy in *Gymnodinium breve* Davis. Science, 137, 988. 17782737 10.1126/science.137.3534.988

[fby043C2] Andeer P. F., Learman D. R., McIlvin M., Dunn J. A. and Hansel C. M. (2015) Extracellular haem peroxidases mediate Mn(II) oxidation in a marine *Roseobacter* bacterium via superoxide production. Environ. Microbiol., 17, 3925–3936. 25923595 10.1111/1462-2920.12893

[fby043C3] Arzul G., Bodennec G., Gentien P., Bornens P. and Crassous M.-P. (1998) The effect of dissolved oxygen on the haemolytic property of *Gymnodinium* ichthyotoxins In Reguera B., Blanco J., Fernandez M. L. and Wyatt T. (eds), Harmful Algae. Xunta de Calicia and Intergovernmental Oceanographic Commision of UNESCO, Vigo, Spain, pp. 611–614.

[fby043C4] Band-Schmidt C. J., Martinez-Lopez A., Bustillos-Guzman J. J., Carreon-Palau L., Morquecho L., Olguin-Monroy N. O., Zenteno-Savin T., Mendoza-Flores A. et al (2012) Morphology, biochemistry, and growth of raphidophyte strains from the Gulf of California. Hydrobiologia, 693, 81–97.

[fby043C5] Bielski B. H. J., Shiue G. G. and Bajuk S. (1980) Reduction of nitro blue tetrazolium by CO_2_^−^ and O_2_^−^ radicals. J. Phys. Chem., 84, 830–833.

[fby043C6] Buetler T. M., Krauskopf A. and Ruegg U. T. (2004) Role of superoxide as a signaling molecule. News Physiol. Sci., 19, 120–123. 15143206 10.1152/nips.01514.2003

[fby043C7] Diaz J. M., Hansel C. M., Voelker B. M., Mendes C. M., Andeer P. F. and Zhang T. (2013) Widespread production of extracellular superoxide by heterotrophic bacteria. Science, 340, 1223–1226. 23641059 10.1126/science.1237331

[fby043C67] Diaz J. M. and Plummer S. (2018) Production of extracellular reactive oxygen species by phytoplankton: past and future directions. J. Plankton Res., 1–12. doi:10.1093/plankt/fby039. 10.1093/plankt/fby039 PMC6247811 30487658

[fby043C8] Dorantes-Aranda J. J., Nichols P. D., Waite T. D. and Hallegraeff G. M. (2013) Strain variability in fatty acid composition of *Chattonella marina* (Raphidophyceae) and its relation to differing ichthyotoxicity toward rainbow trout gill cells. J. Phycol., 49, 427–438. 27008528 10.1111/jpy.12053

[fby043C9] Dorantes-Aranda J. J., Seger A., Mardones J. I., Nichols P. D. and Hallegraeff G. M. (2015) Progress in understanding algal bloom-mediated fish kills: the role of superoxide radicals, phycotoxins and fatty acids. PLoS One, 10, e0133549. 26197230 10.1371/journal.pone.0133549 PMC4509671

[fby043C10] El-Sabaawi R. and Harrison P. J. (2006) Interactive effects of irradiance and temperature on the photosynthetic physiology of the pennate diatom *Pseudo-nitzschia granii* (Bacillariophyceae) from the northeast subarctic Pacific. J. Phycol., 42, 778–785.

[fby043C11] Eng-Wilmot D. L., Hitchcock W. S. and Martin D. F. (1977) Effect of temperature on the proliferation of *Gymnodinium breve* and *Gomphosphaeria aponina*. Mar. Biol., 41, 71–77.

[fby043C12] Flores H. S., Wikfors G. H. and Dam H. G. (2012) Reactive oxygen species are linked to the toxicity of the dinoflagellate *Alexandrium* spp. to protists. Aquat. Microb. Ecol., 66, 199–209.

[fby043C13] Garg S., Rose A. L., Godrant A. and Waite T. D. (2007) Iron uptake by the ichthyotoxic *Chattonella marina* (Raphidophyceae): impact of superoxide generation. J. Phycol., 43, 978–991.

[fby043C14] Godrant A., Rose A. L., Sarthou G. and Waite T. D. (2009) New method for the determination of extracellular production of superoxide by marine phytoplankton using the chemiluminescence probes MCLA and red-CLA. Limnol. Oceanogr. Methods, 7, 682–692.

[fby043C15] Gonzàlez-Flecha B. and Demple B. (1995) Metabolic sources of hydrogen peroxide in aerobically growing *Escherichia coli*. J. Biol. Chem., 270, 13681–13687. 7775420 10.1074/jbc.270.23.13681

[fby043C16] Guillard R. R. L. and Hargraves P. E. (1993) *Stichochrysis immobilis* is a diatom, not a chrysophyte. Phycologia, 32, 234–236.

[fby043C17] Guillard R. R. L. and Ryther J. H. (1962) Studies of marine planktonic diatoms. I. *Cyclotella nana* Hustedt and *Detonula confervacea* Cleve. Can. J. Microbiol., 8, 229–239. 13902807 10.1139/m62-029

[fby043C18] Hansel C. M., Buchwald C., Diaz J. M., Ossolinski J. E., Dyhrman S. T. and Van Mooy B. A. S. (2016) Dynamics of extracellular superoxide production by *Trichodesmium* colonies from the Sargasso Sea. Limnol. Oceanogr., 61, 1188–1200.

[fby043C19] Ishimatsu A., Oda T., Yoshida M. and Ozaki M. (1996) Oxygen radicals are probably involved in the mortality of yellowtail by *Chattonella marina*. Fish. Sci., 62, 836–837.

[fby043C20] Jenkinson I. R. and Arzul G. (2001) Mitigation by cysteine compounds of rheotoxicity, cytotoxicity and fish mortality caused by the dinoflagellates, *Gymnodinium mikimotoi* and *G. cf. maguelonnense* In Hallegraeff G., Bolch C. J. S., Blackburn S. I. and Lewis R. (eds), Harmful Algal Blooms 2000. UNESCO, Paris, pp. 461–464.

[fby043C21] Karlson B., Cusak C. and Bresnan E. (2010) Microscopic and Molecular Methods for Quantitative Phytoplankton Analysis, UNESCO, Paris, France.

[fby043C22] Kawano I., Oda T., Ishimatsu A. and Muramatsu T. (1996) Inhibitory effect of the iron chelator Desferrioxamine (Desferal) on the generation of activated oxygen species by *Chattonella marina*. Mar. Biol., 126, 765–771.

[fby043C23] Kim C. S., Lee S. G., Lee C. K., Kim H. G. and Jung J. (1999a) Reactive oxygen species as causative agents in the ichthyotoxicity of the red tide dinoflagellate *Cochlodinium polykrikoides*. J. Plankton Res., 21, 2105–2115.

[fby043C24] Kim D., Nakamura A., Okamoto T., Komatsu N., Oda T., Iida T., Ishimatsu A. and Muramatsu T. (2000) Mechanism of superoxide anion generation in the toxic red tide phytoplankton *Chattonella marina*: Possible involvement of NAD(P)H oxidase. Biochimica Et Biophysica Acta-General Subjects, 1524, 220–227. 10.1016/s0304-4165(00)00161-6 11113571

[fby043C25] Kim D., Nakamura A., Okamoto T., Komatsu N., Oda T., Ishimatsu A. and Muramatsu T. (1999b) Toxic potential of the raphidophyte *Olisthodiscus luteus*: mediation by reactive oxygen species. J. Plankton Res., 21, 1017–1027.

[fby043C26] Kim D., Nakashima T., Matsuyama Y., Niwano Y., Yamaguchi K. and Oda T. (2007) Presence of the distinct systems responsible for superoxide anion and hydrogen peroxide generation in red tide phytoplankton *Chattonella marina* and *Chattonella ovata*. J. Plankton Res., 29, 241–247.

[fby043C27] Kim D., Oda T., Muramatsu T., Kim D., Matsuyama Y. and Honjo T. (2002) Possible factors responsible for the toxicity of *Cochlodinium polykrikoides*, a red tide phytoplankton. Comp. Biochem. Physiol. C Toxicol. Pharmacol., 132, 415–423. 12223197 10.1016/s1532-0456(02)00093-5

[fby043C28] Kustka A. B., Shaked Y., Milligan A. J., King D. W. and Morel F. M. M. (2005) Extracellular production of superoxide by marine diatoms: Contrasting effects on iron redox chemistry and bioavailability. Limnol. Oceanogr., 50, 1172–1180.

[fby043C29] Li X., Liu T., Wang K. and Waite T. D. (2015) Light-induced extracellular electron transport by the marine raphidophyte *Chattonella marina*. Environ. Sci. Technol., 49, 1392–1399. 25569116 10.1021/es503511m

[fby043C30] Liu W., Au D. W. T., Anderson D. M., Lam P. K. S. and Wu R. S. S. (2007) Effects of nutrients, salinity, pH and light:dark cycle on the production of reactive oxygen species in the alga *Chattonella marina*. J. Exp. Mar. Bio. Ecol., 346, 76–86.

[fby043C31] Mardones J. I., Dorantes-Aranda J. J., Nichols P. D. and Hallegraeff G. M. (2015) Fish gill damage by the dinoflagellate *Alexandrium catenella* from Chilean fjords: synergistic action of ROS and PUFA. Harmful Algae, 49, 40–49.

[fby043C32] Marshall J. A., de Salas M., Oda T. and Hallegraeff G. (2005a) Superoxide production by marine microalgae: I. survey of 37 species from 6 classes. Mar. Biol., 147, 533–540.

[fby043C33] Marshall J. A., Nichols P. D., Hamilton B., Lewis R. J. and Hallegraeff G. M. (2003) Ichthyotoxicity of *Chattonella marina* (Raphidophyceae) to damselfish (*Acanthochromis polycanthus*): the synergistic role of reactive oxygen species and free fatty acids. Harmful Algae, 2, 273–281.

[fby043C34] Marshall J. A., Ross T., Pyecroft S. and Hallegraeff G. (2005b) Superoxide production by marine microalgae: II. towards understanding ecological consequences and possible functions. Mar. Biol., 147, 541–549.

[fby043C35] Miller W. L. and Kester D. R. (1988) Hydrogen peroxide measurement in seawater by (para-hydroxylphenyl)acetic acid dimerization. Anal. Chem., 60, 2711–2715.

[fby043C36] Milligan A. J. and Cosper E. M. (1997) Growth and photosynthesis of the ‘brown tide’ microalga *Aureococcus anophagefferens* in subsaturating constant and fluctuating irradiance. Mar. Ecol. Prog. Ser., 153, 67–75.

[fby043C37] Mittler R., Vanderauwera S., Suzuki N., Miller G., Tognetti V. B., Vandepoele K., Gollery M., Shulaev V. et al (2011) ROS signaling: the new wave? Trends Plant Sci., 16, 300–309. 21482172 10.1016/j.tplants.2011.03.007

[fby043C38] Mooney B. D., Dorantes-Aranda J. J., Place A. R. and Hallegraeff G. M. (2011) Ichthyotoxicity of gymnodinioid dinoflagellates: PUFA and superoxide effects in sheepshead minnow larvae and rainbow trout gill cells. Mar. Ecol. Prog. Ser., 426, 213–224.

[fby043C39] Oda T., Ishimatsu A., Shimada M., Takeshita S. and Muramatsu T. (1992) Oxygen-radical-mediated toxic effects of the red tide flagellate *Chattonella marina* on *Vibrio alginolyticus*. Mar. Biol., 112, 505–509.

[fby043C40] Oda T., Moritomi J., Kawano I., Hamaguchi S., Ishimatsu A. and Muramatsu T. (1995) Catalase-induced and superoxide dismutase-induced morphological changes and growth inhibition in the red tide phytoplankton *Chattonella marina*. Biosci. Biotechnol. Biochem., 59, 2044–2048.

[fby043C41] Oda T., Nakamura A., Okamoto T., Ishimatsu A. and Muramatsu T. (1998) Lectin-induced enhancement of superoxide anion production by red tide phytoplankton. Mar. Biol., 131, 383–390.

[fby043C42] Oda T., Nakamura A., Shikayama M., Kawano I., Ishimatsu A. and Muramatsu T. (1997) Generation of reactive oxygen species by raphidophycean phytoplankton. Biosci. Biotechnol. Biochem., 61, 1658–1662. 9362113 10.1271/bbb.61.1658

[fby043C43] Portune K. J., Cary S. C. and Warner M. E. (2010) Antioxidant enzyme response and reactive oxygen species production in marine raphidophytes. J. Phycol., 46, 1161–1171.

[fby043C44] Pullin M. J., Bertilsson S., Goldstone J. V. and Voelker B. M. (2004) Effects of sunlight and hydroxyl radical on dissolved organic matter: Bacterial growth efficiency and production of carboxylic acids and other substrates. Limnol. Oceanogr., 49, 2011–2022.

[fby043C45] Roe K. L. and Barbeau K. A. (2014) Uptake mechanisms for inorganic iron and ferric citrate in *Trichodesmium erythraeum* IMS101. Metallomics, 6, 2042–2051. 25222699 10.1039/c4mt00026a

[fby043C46] Rose A. L. (2012) The influence of extracellular superoxide on iron redox chemistry and bioavailability to aquatic microorganisms. Front. Microbiol., 3, 124. 22514548 10.3389/fmicb.2012.00124 PMC3323869

[fby043C47] Rose A. L., Salmon T. P., Lukondeh T., Neilan B. A. and Waite T. D. (2005) Use of superoxide as an electron shuttle for iron acquisition by the marine cyanobacterium *Lyngbya majuscula*. Environ. Sci. Technol., 39, 3708–3715. 15952376 10.1021/es048766c

[fby043C48] Rose A. L., Webb E. A., Waite T. D. and Moffett J. W. (2008) Measurement and implications of nonphotochemically generated superoxide in the equatorial Pacific Ocean. Environ. Sci. Technol., 42, 2387–2393. 18504970 10.1021/es7024609

[fby043C49] Saragosti E., Tchernov D., Katsir A. and Shaked Y. (2010) Extracellular production and degradation of superoxide in the coral *Stylophora pistillata* and cultured *Symbiodinium*. PLoS One, 5, e12508. 20856857 10.1371/journal.pone.0012508 PMC2939047

[fby043C50] Saran M. (2003) To what end does nature produce superoxide? NADPH oxidase as an autocrine modifier of membrane phospholipids generating paracrine lipid messengers. Free Radic. Res., 37, 1045–1059. 14703794 10.1080/10715760310001594631

[fby043C51] Schneider R. J., Roe K. L., Hansel C. M. and Voelker B. M. (2016) Species-level variability in extracellular production rates of reactive oxygen species by diatoms. Front. Chem., 4, 5. 27066475 10.3389/fchem.2016.00005 PMC4812844

[fby043C52] Shikata T., Nagasoe S., Matsubara T., Yoshikawa S., Yamasaki Y., Shimasaki Y., Oshima Y., Jenkinson I. R. et al (2008) Factors influencing the initiation of blooms of the raphidophyte *Heterosigma akashiwo* and the diatom *Skeletonema costatum* in a port in Japan. Limnol. Oceanogr., 53, 2503–2518.

[fby043C53] Siciliano S. D., O’Driscoll N. J. and Lean D. R. S. (2002) Microbial reduction and oxidation of mercury in freshwater lakes. Environ. Sci. Technol., 36, 3064–3068. 12141483 10.1021/es010774v

[fby043C54] Skeen A. R., Tomas C. R. and Cooper W. J. (2004) The production of hydrogen peroxide by *Heterosigma akashiwo* under varying N:P ratios In Steidinger K. A., Landsberg J. H., Tomas C. R. and Vargo G. A. (eds), Harmful Algae 2002. Florida Fish and Wildlife Conservation Commission, Florida Institute of Oceanography, and Intergovernmental Oceanographic Commission of UNESCO, St. Petersburg, FL, USA, pp. 77–79.

[fby043C55] Smayda T. J. (2008) Complexity in the eutrophication-harmful algal bloom relationship, with comment on the importance of grazing. Harmful Algae, 8, 140–151.

[fby043C56] Tang Y. Z. and Gobler C. J. (2009a) Characterization of the toxicity of *Cochlodinium polykrikoides* isolates from Northeast US estuaries to finfish and shellfish. Harmful Algae, 8, 454–462.

[fby043C57] Tang Y. Z. and Gobler C. J. (2009b) *Cochlodinium polykrikoides* blooms and clonal isolates from the northwest Atlantic coast cause rapid mortality in larvae of multiple bivalve species. Mar. Biol., 156, 2601–2611.

[fby043C58] Tang Y. Z. and Gobler C. J. (2010) Allelopathic effects of *Cochlodinium polykrikoides* isolates and blooms from the estuaries of Long Island, New York, on co-occurring phytoplankton. Mar. Ecol. Prog. Ser., 406, 19–31.

[fby043C59] Twiner M. J., Dixon S. J. and Trick C. G. (2001) Toxic effects of *Heterosigma akashiwo* do not appear to be mediated by hydrogen peroxide. Limnol. Oceanogr., 46, 1400–1405.

[fby043C60] Twiner M. J. and Trick C. G. (2000) Possible physiological mechanisms for production of hydrogen peroxide by the ichthyotoxic flagellate *Heterosigma akashiwo*. J. Plankton Res., 22, 1961–1975.

[fby043C61] Wilson W. B. and Collier A. (1955) Preliminary notes on the culturing of *Gymnodinium breve* Davis. Science, 121, 394–395. 17837228 10.1126/science.121.3142.394

[fby043C62] Woo S. P. S., Liu W. H., Au D. W. T., Anderson D. M. and Wu R. S. S. (2006) Antioxidant responses and lipid peroxidation in gills and erythrocytes of fish (*Rhabdosarga sarba*) upon exposure to *Chattonella marina* and hydrogen peroxide: Implications on the cause of fish kills. J. Exp. Mar. Bio. Ecol., 336, 230–241.

[fby043C63] Yamaguchi M., Imai I. and Honjo T. (1991) Effects of temperature, salinity and irradiance on the growth rates of the noxious red tide flagellates *Chattonella antiquia* and *C. marina* (Raphidophyceae). Nippon Suisan Gakkaishi, 57, 1277–1284.

[fby043C64] Yang C. Z., Albright L. J. and Yousif A. N. (1995) Oxygen-radical-mediated effects of the toxic phytoplankter *Heterosigma carterae* on juvenile rainbow trout *Oncorhynchus mykiss*. Dis. Aquat. Organ., 23, 101–108.

[fby043C65] Zhang T., Diaz J. M., Brighi C., Parsons R. J., McNally S., Apprill A. and Hansel C. M. (2016) Dark production of extracellular superoxide by the coral *Porites astreoides* and representative symbionts. Front. Mar. Sci., 3, 232.

[fby043C66] Zinser E. R. (2018) The microbial contribution to reactive oxygen species dynamics in marine ecosystems. Environ. Microbiol. Rep., 10, 412–427. 29411545 10.1111/1758-2229.12626

